# TOR balances plant growth and cold tolerance by orchestrating amino acid-derived metabolism in tomato

**DOI:** 10.1093/hr/uhae253

**Published:** 2024-09-05

**Authors:** Zihao Li, Lin Yang, Yanni Wu, Ran Zhang, Sen Yu, Liwen Fu

**Affiliations:** Shanghai Collaborative Innovation Center of Agri-Seeds, School of Agriculture and Biology, Shanghai Jiao Tong University, Shanghai 200240, China; Joint Center for Single Cell Biology, School of Agriculture and Biology, Shanghai Jiao Tong University, Shanghai 200240, China; Shanghai Collaborative Innovation Center of Agri-Seeds, School of Agriculture and Biology, Shanghai Jiao Tong University, Shanghai 200240, China; Joint Center for Single Cell Biology, School of Agriculture and Biology, Shanghai Jiao Tong University, Shanghai 200240, China; Shanghai Collaborative Innovation Center of Agri-Seeds, School of Agriculture and Biology, Shanghai Jiao Tong University, Shanghai 200240, China; Joint Center for Single Cell Biology, School of Agriculture and Biology, Shanghai Jiao Tong University, Shanghai 200240, China; Shanghai Collaborative Innovation Center of Agri-Seeds, School of Agriculture and Biology, Shanghai Jiao Tong University, Shanghai 200240, China; Joint Center for Single Cell Biology, School of Agriculture and Biology, Shanghai Jiao Tong University, Shanghai 200240, China; Shanghai Collaborative Innovation Center of Agri-Seeds, School of Agriculture and Biology, Shanghai Jiao Tong University, Shanghai 200240, China; Joint Center for Single Cell Biology, School of Agriculture and Biology, Shanghai Jiao Tong University, Shanghai 200240, China; Shanghai Collaborative Innovation Center of Agri-Seeds, School of Agriculture and Biology, Shanghai Jiao Tong University, Shanghai 200240, China; Joint Center for Single Cell Biology, School of Agriculture and Biology, Shanghai Jiao Tong University, Shanghai 200240, China

## Abstract

The target of rapamycin (TOR) kinase is a central signaling hub that plays a crucial role in precisely orchestrating plant growth, development, and stress responses. This suggests that TOR is intricately involved in maintaining the balance between plant growth and stress responses. Nevertheless, despite the observed effects, the specific mechanisms through which TOR operates in these processes remain obscure. In this study, we investigated how the tomato (*Solanum lycopersicum*) TOR (*Sl*TOR) affects plant growth and cold responses. We demonstrated that *Sl*TOR inhibition transcriptionally primes cold stress responses, consequently enhancing tomato cold tolerance. A widely targeted metabolomics analysis revealed the disruption of amino acid metabolism homeostasis under cold stress upon *Sl*TOR inhibition, which led to the accumulation of two important cryoprotective metabolites: salicylic acid (SA) and putrescine (Put). Next, we discovered *Sl*PGH1 (2-PHOSPHO-D-GLYCERATE HYDRO-LYASE 1) as a direct substrate of *Sl*TOR. Inhibiting *Sl*TOR led to increased *SlCBF1* (*C-REPEAT-BINDING FACTOR 1*) expression via *Sl*PGH1, potentially triggering the activation of cold-responsive genes and subsequent metabolic alterations. Our study provides a mechanistic framework that elucidates how *Sl*TOR modulates amino acid-related metabolism to enhance tomato cold tolerance, which sheds light on the complex interplay between growth and stress responses orchestrated by TOR.

## Introduction

In response to adverse environmental factors like cold, heat, drought, salinity, limited nutrients, and various pathogens, plants redirect their limited resources from growth toward stress responses, which enables them to survive under unfavorable conditions, albeit at the expense of growth [1]. Such a trade-off between growth and stress responses plays a significant role in determining the fitness and survival of plants. Therefore, comprehending the molecular mechanisms driving this trade-off is crucial.

Target of rapamycin (TOR) is an evolutionarily conserved serine/threonine protein kinase found in eukaryotes, which exerts its function in complex forms [2]. In yeast and mammals, two main TOR complexes have been characterized [3]. Despite the high amino acid sequence similarity with mTOR, only one TOR complex has been identified in plants, comprising LST8, Raptor, and TOR itself [4]. TOR is a central signaling hub in plants, integrating various internal and external signals to finely orchestrate nearly every aspect of plant growth and development [5–12]. Furthermore, mounting evidence indicates that TOR also significantly regulates plants’ responses to diverse abiotic and biotic stresses [2, 13–16]. Different researchers have carefully examined the TOR-guided growth–defense trade-offs due to TOR’s essential roles in both processes [13, 17]. The underlying mechanism for TOR-steered growth–defense trade-offs, however, remains elusive. The transition between growth and defense involves significant transcriptional, translational, and metabolic changes to reallocate energy and nutrients between these two biological processes. It is yet to be tested whether and how TOR functions in this process.

The frequency of low-temperature events has significantly increased in recent years due to unpredictable climate changes [18]. Tomato, an economically significant global vegetable crop originating from tropical and subtropical regions, is particularly vulnerable to low temperatures throughout all stages of its growth and development [19, 20]. Plants have developed sophisticated mechanisms to promptly detect and respond to cold stress. Cold stress is primarily sensed by the cell membrane, causing changes in membrane fluidity, lipid composition, and function of membrane-bound proteins, finally activating downstream cold stress responses [18]. Cold stress responses involve the central role of C-Repeat-Binding Factors (CBFs). Under cold stress, CBFs trigger a large-scale transcriptional reprogramming, particularly inducing the cold-regulated genes modulating cryoprotective metabolite accumulation, which enhance plant cold tolerance [18]. TOR’s role in *Arabidopsis* cold stress responses has been carefully examined by some independent studies [21–23]. Cold stress dynamically affects TOR activity. Initially, TOR activity is inhibited rapidly and transiently; however, prolonged cold stress leads to an enhancement of TOR activity [21, 23]. TOR-regulated cold responses are intricate. For instance, the FERONIA-ROP2-TOR axis has been demonstrated to promote root hair growth in response to low temperatures [23]. Estradiol-induced *tor* mutant was reported to exhibit increased cold tolerance [21]. Conversely, another independent research suggested that TOR positively regulates cold stress responses [22]. These findings underscore the complex and multifaceted involvement of TOR in cold stress responses.

Here, we reveal that inhibition of *Sl*TOR induces numerous cold-regulated genes while decreasing the expression of growth-related genes, highlighting *Sl*TOR’s pivotal role in balancing cold stress responses and growth. Our findings suggest that cold stress promptly and transiently suppresses TOR activity, and blocking TOR function amplifies plants’ tolerance to cold. Metabolomics analysis revealed that inhibiting TOR during cold stress disrupts the amino acids biosynthesis and promotes the production of salicylic acid (SA), putrescine (Put), and various flavonoids, contributing to enhanced cold stress tolerance. Then, we identified 2-phospho-D-glycerate hydro-lyase 1 (*Sl*PGH1) as a direct substrate of *Sl*TOR. Inhibiting *Sl*TOR increased *SlCBF1* expression through *Sl*PGH1, potentially activating cold-responsive genes and inducing metabolic reprogramming. This investigation delineates how *Sl*TOR orchestrates amino acid-related metabolism to bolster tomato cold tolerance and deepens our comprehension of the TOR-mediated growth–defense trade-off.

## Results

### *Sl*TOR promotes growth and drives large-scale transcription reprogramming

**Figure 1:**
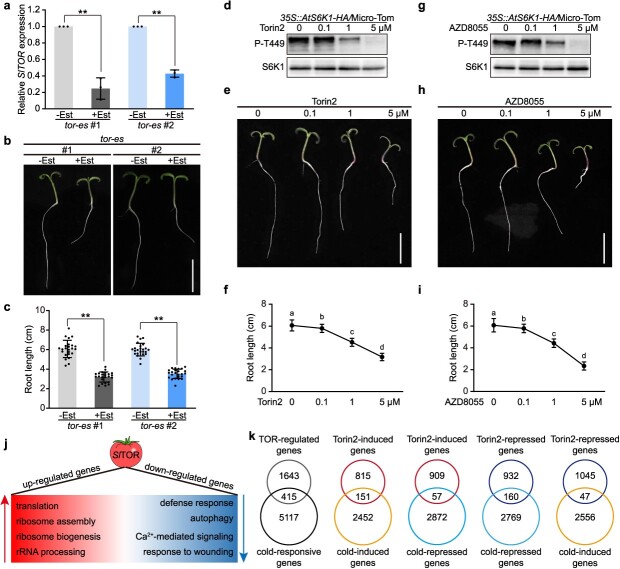
*Sl*TOR promotes growth and drives large-scale transcription reprogramming in tomato. (a) RT-qPCR analysis of the transcript level of *SlTOR* in 7-day-old estradiol-inducible RNA interference *tor-es* seedlings with or without 10-μM Est treatment at the time of germination. Mean ± SD, *n* = 3 biological replicates. (b) *tor-es* tomato plants exhibited severe growth arrest under estradiol (Est) treatment. Est (10 μM) was added at the time of germination for 7 days. Scale bar = 2 cm. (c) Quantification of the root length of (b). Data were analyzed from 24 seedlings for each treatment from three experiments, and expressed as mean ± SD. (d) and (g) Western blot analysis of *Sl*TOR kinase activity in *35S::AtS6K1-HA/*Micro-Tom transgenic seedlings, based on phosphorylation of Thr449 on S6K1 (P-T449, a specific phosphorylation site of TOR kinase on S6K1) after treatment with indicated concentrations of Torin2 (d) or AZD8055 (g). (e) and (h) Torin2 (e) or AZD8055 (h) retarded tomato seedling growth. Tomato seeds were germinated on 1/2 MS medium with different concentrations of Torin2 or AZD8055 for 7 days. Scale bars = 2 cm. (f) and (i) Quantifications of the root length of (e) and (h). Data were analyzed from 30 seedlings for each treatment from three experiments, and expressed as mean ± SD (j) An overview of biological processes regulated by *Sl*TOR signaling as determined by RNA-seq analysis. 10-day-old tomato seedlings were treated with or without 5 μM Torin2 for 2 h. The RNA-seq results indicated that *Sl*TOR promoted growth-related biological processes while suppressing stress responses. (k) Comparisons between the TOR-regulated and the cold-regulated transcriptomes. Statistical significance in (a) and (c) was determined by two-sided Student’s *t*-test. ^**^, *P* < 0.01. Statistical significance in (f) and (i) was determined by one-way ANOVA with Tukey’s *post hoc* test. Lowercase letters indicate statistically significant differences between the mean values (*P* < 0.05).

To investigate the function of *Sl*TOR in tomato (Micro-Tom) growth, we generated estradiol-inducible RNA interference *tor* transgenic tomato plants (*tor-es*). These *tor-es* plants were treated with 10 μM estradiol in 1/2 MS medium, leading to the repression of *SlTOR* transcripts (Fig. 1a). Subsequently, we observed inhibited root elongation in *tor-es* seedlings 7 days after germination (Fig. 1b–c). Different chemical inhibitors are widely used to study TOR’s function in plants [4, 24]. We first checked these inhibitors’ effects on *Sl*TOR activity. *At*TOR-induced phosphorylation of *At*S6K1 is a conserved indicator of endogenous *At*TOR activity [25]. Considering the conservation between *At*TOR and *Sl*TOR [24], we overexpressed *AtS6K1* in tomato to monitor *Sl*TOR activity. Gradient concentrations of Torin2 or AZD8055 (0, 0.1, 1, and 5 μM) were applied to the transgenic seedlings. Gradually reduced phosphorylated *At*S6K1 protein was detected in the immunoblot analysis post-TOR inhibitor treatment, and 5 μM Torin2 or AZD8055 effectively blocked *Sl*TOR activity (Fig. 1d and 1g, Fig. S1). Next, we examined the impact of these TOR inhibitors on tomato growth. Wild-type (WT) tomato seeds were germinated on 1/2 MS medium with Torin2 or AZD8055 (0, 0.1, 1, and 5 μM). After 7 days, we noted a gradual inhibition of root growth in tomato seedlings grown on TOR inhibitor-containing medium (Fig. 1e-f, 1h-i). Notably, 5 μM Torin2 or AZD8055 significantly impeded tomato root growth, consistent with the *Sl*TOR activity measurements (Fig. 1d–i, Fig. S1).

RNA-seq analysis was further conducted using total RNA extracted from 10-day-old tomato seedlings subjected to 2-h treatment with 5 μM Torin2 to investigate *Sl*TOR’s function. The analysis identified 2058 differentially expressed genes (DEGs) (FC > 2, FDR < 0.05) in Torin2-treated seedlings compared to the control group, with 966 genes upregulated and 1092 genes downregulated (Table S2). Gene ontology (GO) term enrichment analysis was then performed to investigate the *Sl*TOR-modulated biological processes. A total of 66.9% of the DEGs (1377 out of 2058) were associated with significantly enriched GO terms (Table S3). Specifically, the GO term enrichment analysis revealed that *Sl*TOR activation triggered multiple growth-promoting processes, including translation, ribosome assembly, ribosome biogenesis, and rRNA processing, while concurrently inhibiting defense-related mechanisms such as defense response, autophagy, Ca^2+^-mediated signaling, and wounding response (Fig. 1j, Table S3). The functional analysis suggests that *Sl*TOR could potentially operate as a critical regulator balancing growth and defense in tomato.

Examining the 966 Torin2-induced genes, several significant cold-regulated genes, including *SlCAMTA3* (*CALMODULIN-BINDING TRANSCRIPTION ACTIVATOR 3*, Solyc04g056270.4), *SlCBF1* (Solyc03g026280.3), and *SlZAT10* (*ZINC FINGER of Arabidopsis thaliana 10*, Solyc01g107170.2), were identified. This discovery prompted us to compare the 2058 *Sl*TOR-regulated genes with a set of previously reported 5532 cold-regulated genes [26]. This investigation unveiled a noteworthy overlap between these two gene sets, with 415 DEGs under Torin2 treatment also showing differential expression during cold stress (Fig. 1k, Table S4). Among the 415 overlapping genes, 151 genes were enhanced by both Torin2 treatment and cold stress, and 160 genes were repressed by both Torin2 treatment and cold stress (Table S4). This indicates that 311 (151 plus 160) genes showed similar expression patterns, accounting for 75% of the 415 total overlapping genes. Furthermore, a higher number of overlapping genes was observed between Torin2-induced and cold-induced genes compared to the overlap between Torin2-induced and cold-repressed genes; and a similar pattern was also noted in the comparisons between Torin2-repressed and cold-repressed genes, as well as between Torin2-repressed and cold-induced genes (Fig. 1k, Table S4). The findings suggest that *Sl*TOR may have a negative regulatory effect on cold stress responses (Fig. 1k).

### *Sl*TOR negatively regulates cold stress responses

The transcriptome analysis indicated that *Sl*TOR might negatively regulate cold stress responses. To explore this further, we subjected 4-week-old tomato plants to a 2-h pretreatment with 10 μM Torin2 or AZD8055 through foliar spraying, followed by a 4°C treatment. The assessment of cell viability using electrolyte leakage analysis demonstrated that mock-treated plants exhibited a significant cell death phenotype at 4°C compared to the Torin2- and AZD8055-treated plants as early as 1 day post-treatment (Fig. S2a). With increasing treatment duration, the plants with *Sl*TOR inactivation maintained a reasonable level of cold tolerance even after enduring a 10-day cold stress period (Fig. S2a). As inhibition of *Sl*TOR induces the expression of cold-regulated genes and enhances cold tolerance, we speculated that cold stress might inhibit *Sl*TOR activity to trigger cold stress responses, serving as a strategy for plants to cope with cold stress. To investigate this further, transgenic tomato plants overexpressing *AtS6K1* were exposed to 4°C for various durations. Surprisingly, the cold treatment rapidly and temporarily inhibited *Sl*TOR activity, determined by the level of phosphorylated *At*S6K1 protein (Fig. S2b–c). However, *Sl*TOR activity recovered following 2 h of cold treatment (Fig. S2b–c). This dynamic response indicates that short-term *Sl*TOR inhibition is an early response to cold stress. Nevertheless, active *Sl*TOR is still essential for tomato plants during long-term cold exposure. Meanwhile, treating with Torin2 at 25°C or pretreating with Torin2 for 2 h before exposure to 4°C via foliar spray effectively inhibited TOR 2 h post-inhibitor treatment (Fig. S2d–g).

**Figure 2:**
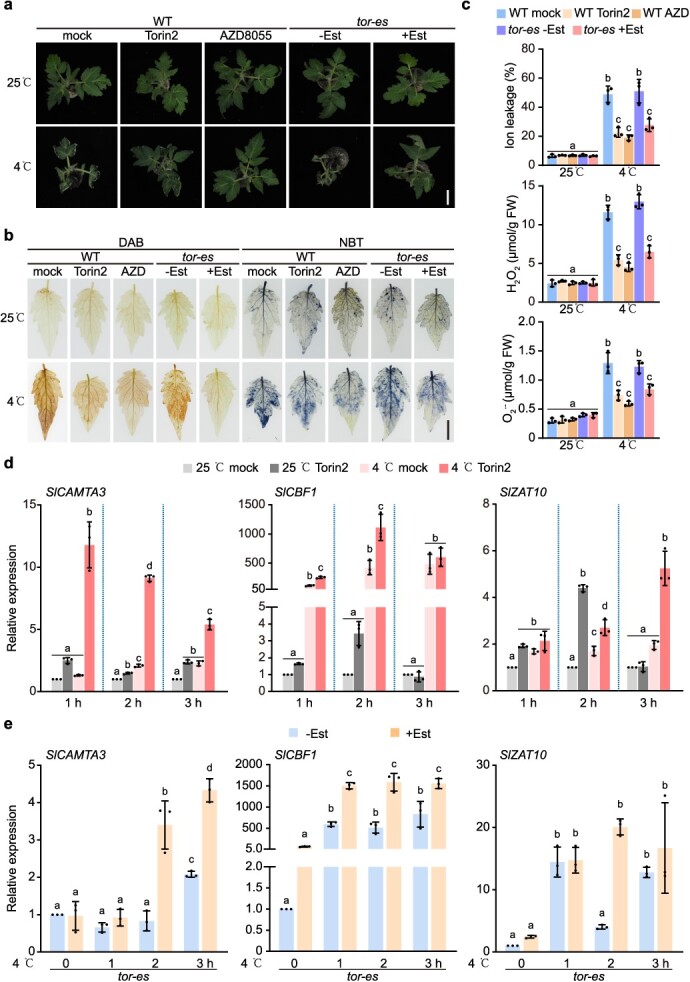
*Sl*TOR negatively regulates cold stress responses. (a) Torin2 or AZD8055 treatment enhanced 4-week-old WT tomatoes’ cold tolerance, while Est-treated 4-week-old *tor-es* plants also showed elevated cold tolerance. Representative images were captured. Scale bar = 2 cm. (b) TOR inhibitor-treated WT plants or Est-treated *tor-es* plants exhibited alleviated H_2_O_2_ and O_2_^• –^ release, as indicated by DAB and NBT staining, respectively. Representative images were captured. Scale bar = 1 cm. (c) Quantification analysis of ion leakage, H_2_O_2_ and O_2_^• –^ under different treatments. Data were analyzed from 3 experiments, and expressed as mean ± SD. (d) RT-qPCR analysis of the transcript levels of *SlCAMTA3*, *SlCBF1*, and *SlZAT10* in 4-week-old WT tomato plants under different treatments at indicated times. Mean ± SD, *n* = 3 biological replicates. (e) RT-qPCR analysis of the transcript levels of *SlCAMTA3*, *SlCBF1*, and *SlZAT10* in 4-week-old *tor-es* tomato plants under cold treatment at indicated times. Mean ± SD, *n* = 3 biological replicates. One week before treatment, the *tor-es* seedlings were watered with 10 μM Est to silence *SlTOR*, and then were subject to 4°C cold treatment for different durations. The *tor-es* seedlings without Est treatment were used as control seedlings. Statistical significance in (c), (d) and (e) was determined by one-way ANOVA with Tukey’s *post hoc* test. For (d), statistical significance was calculated based on expression data at indicated time points, respectively. Lowercase letters indicate statistically significant differences between the mean values (*P* < 0.05).

Based on the above results, the macroscopic differences resulting from *Sl*TOR inhibition or *SlTOR* knockdown were further analyzed at the initial stage of wilting in tomato plants (1 day post-4°C treatment, Fig. S2a). Specifically, 1 day after exposure to cold stress, we observed a more severely wilted state of the WT mock-treated plants and non-estradiol-treated *tor-es* plants compared to *Sl*TOR-repressed plants by chemical inhibitors or RNAi (Fig. 2a). To assess the wilted and necrotic phenotypes quantitatively, electrolyte leakage analysis and examination of hydrogen peroxide (H_2_O_2_) and superoxide anion (O_2_^• –^) levels were performed (Fig. 2b–c). The levels of intercellular free ions, H_2_O_2_, and O_2_^• –^ were significantly lower in TOR-dysfunctional plants compared to control plants at 4°C, but showed no significant differences at 25°C (Fig. 2b–c). Furthermore, RNA-seq data revealed that the suppression of *Sl*TOR led to transcriptional reprogramming of numerous cold-regulated genes (Fig. 1k). To investigate the involvement of *Sl*TOR in cold-induced transcriptional reprogramming, leaf samples with the aforementioned treatments were collected to examine the expression levels of three key *Sl*TOR-regulated cold-responsive genes (*SlCAMTA3*, *SlCBF1*, and *SlZAT10*). Consistent with the RNA-seq findings, the inhibition of *Sl*TOR resulted in the upregulation of these genes at 25°C (Fig. 2d–e). Additionally, these cold-regulated genes exhibited greater induction in *Sl*TOR-deficient plants compared to the control plants under chilling conditions (Fig. 2d–e). Our comprehensive results indicate that *Sl*TOR functions as a negative regulator of cold stress responses in tomato.

**Figure 3:**
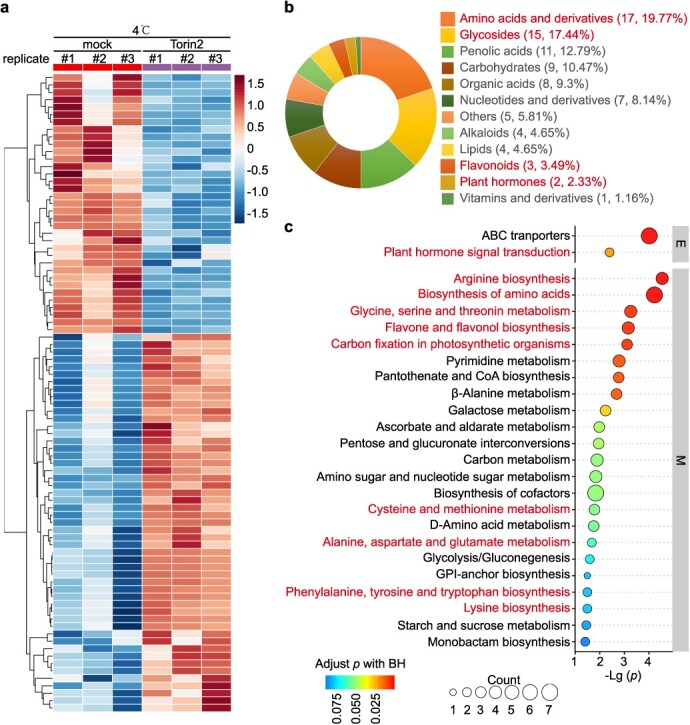
Widely targeted metabolomics analysis reveals a contrasting accumulation of metabolites between Torin2-treated and mock-treated tomato plants during cold stress. (a) The heat map illustrates DAMs between mock and Torin2-treated tomato plants under cold conditions. Each row represents a single metabolite. (b) The pie chart exhibits the biochemical classification of the DAMs. (c) KEGG pathway enrichment analysis for DAMs.

**Figure 4:**
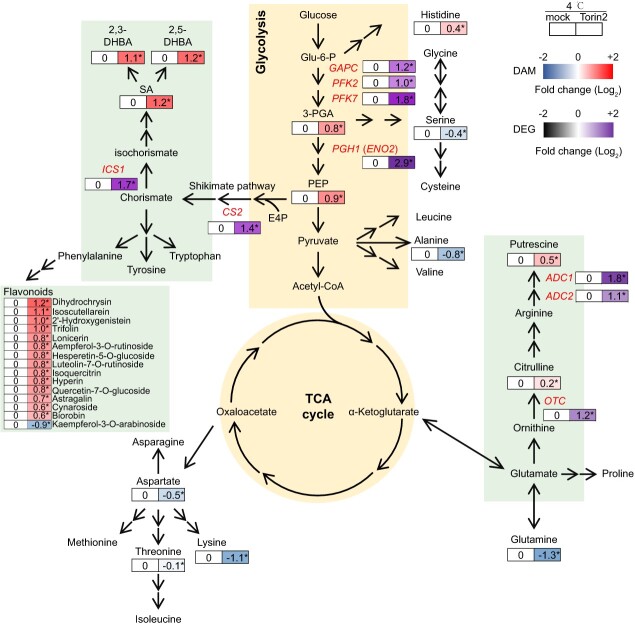
A schematic diagram of the amino acid-related metabolic pathway highlighting Torin2-modulated DEGs and DAMs during cold stress. Torin2 treatment induced significant metabolic alternations under 4°C conditions. Briefly, biosynthesis of amino acids was inhibited, while SA, Put, and flavonoid biosynthesis were elevated. Data are represented as log_2_ FC of Torin2 treatment compared with mock treatment. The expression patterns of the genes encoding the core enzymes in metabolic pathways, as indicated by the DEGs, align well with the Torin2-induced metabolite alterations. Data are represented as log_2_ FC of Torin2 treatment compared with mock treatment. Additional details can be found in Table S7 and Fig. S7. Statistical significance for both DAMs and DEGs was assessed using a two-sided Student’s *t*-test, denoted by an asterisk (*) next to the log_2_ FC value (*P* < 0.05).

### *Sl*TOR orchestrates significant metabolic reprogramming under cold stress

Previous studies have shown that TOR mediates significant metabolic changes in *Arabidopsis* and *Chlamydomonas reinhardtii* [6–8, 27–31]. As various metabolites are also crucial for plant cold stress responses, we tested TOR-regulated metabolic reprogramming for the putative mechanism of TOR-mediated cold stress responses. Specifically, we conducted a widely targeted metabolome profiling of tomato plants within the plants subjected to a 2-h Torin2 pretreatment and control plants exposed to 4°C and 25°C for 24 h to explore the metabolite changes affected by *Sl*TOR. The partial least squares discriminant analysis (PLS-DA) indicates the robust reproducibility of the metabolite model (Fig. S3a–b).

The transition from 25°C to 4°C resulted in 69 differentially accumulated metabolites (DAMs, *P* < 0.05) with 40 being upregulated and 29 downregulated (Fig. S4, Table S5). Under 25°C, we identified 31 DAMs (*P* < 0.05) between Torin2- and mock-treated tomato plants (Fig. S4, Table S6). Among these, 17 DAMs showed upregulation while 14 were downregulated. Torin2 pretreatment at 4°C revealed a broader spectrum of metabolic changes, identifying a total of 86 DAMs (*P* < 0.05). Of these, 51 were upregulated and 35 were downregulated (Fig. S4, Fig. 3a, and Table S7). These distinct metabolic patterns were clustered and their scaled intensity visualized in a hierarchical cluster heat map (Fig. S4 and Fig. 3a). We conducted comparisons among cold-induced DAMs, DAMs triggered by Torin2 at 25°C, and DAMs induced by Torin2 treatment at 4°C. While there were shared DAMs between the groups that underwent cold treatment alone and the group treated with Torin2 at 4°C, as well as between the Torin2 treatment at 25°C and Torin2 treatment at 4°C, it is noteworthy that Torin2 pretreatment at 4°C led to the emergence of a substantial number of distinct DAMs (Fig. S5 and Table S8).

We then checked the 86 DAMs upon Torin2 pretreatment at 4°C. The clustering heat map in Fig. 3a elucidated the metabolite accumulation patterns. These DAMs were classified into 12 classes, with the top three classes being ‘Amino acids and derivatives’ (17, 19.77%), ‘Glycosides’ (15, 17.44%), and ‘Phenolic acids’ (11, 12.79%) as shown in Fig. 3b. Intriguingly, the presence of ‘Flavonoids’ and ‘Plant hormones’, known for regulating stress responses [32, 33], was also observed in Fig. 3b. Furthermore, KEGG pathway enrichment analysis revealed the significant enrichment of several pathways including amino acid biosynthesis pathways, ‘Flavone and flavonol biosynthesis’, ‘Carbon fixation in photosynthetic organisms’, and ‘Plant hormone signal transduction’ (Fig. 3c and Table S9). Therefore, these highlighted pathways and associated metabolites might be potential contributors to enhancing tomato cold tolerance upon *Sl*TOR inhibition.

### *Sl*TOR orchestrated amino acid-derived metabolism to mediate the balance between growth and defense under cold stress condition

Upon Torin2 treatment at 4°C, most amino acid biosynthesis pathways were repressed as revealed by KEGG enrichment and differential abundance score (DA score) analysis (Fig. S6 and Table S9). However, previous research indicated that disruptions in TOR under normal temperatures have been associated with increased levels of amino acids [28, 30, 31]. Interestingly, the metabolites involved in ‘Phenylalanine, tyrosine, and tryptophan biosynthesis’ pathway were accumulated (Fig. S6, Fig. 4, and Table S9).

Enhancing the ‘Phenylalanine, tyrosine, and tryptophan biosynthesis’ pathway amid the repression of most amino acid biosynthesis pathways prompted a detailed exploration of the metabolic changes in this specific pathway (Fig. S6). In plants, glycolysis intermediates, specifically 3-phosphoglycerate (3-PGA) and phosphoenolpyruvate (PEP), serve as the carbon skeleton for amino acid biosynthesis (Fig. 4). PEP, in conjunction with the pentose phosphate pathway intermediate D-erythrose 4-phosphate (E4P), undergoes a sequence of plastidial enzymatic reactions in the shikimate pathway to produce chorismite (Fig. 4), a crucial precursor for the aromatic amino acids-phenylalanine, tyrosine, and tryptophan. Moreover, flavonoids, recognized for their pivotal roles in plant defense responses, are synthesized from phenylalanine through the phenylpropanoid pathway (Fig. 4) [34, 35]. Notably, aside from amino acid synthesis, chorismite can be further converted to isochorismate, culminating in the production of SA and its derivatives (Fig. 4) [36].

After a Torin2 pretreatment, the glycolysis intermediates 3-PGA and PEP exhibited a marked increase at 4°C in our metabolic profile (Fig. 4). This increase was accompanied by a notable downregulation of serine and alanine, which are known to acquire carbon skeletons from glycolysis intermediates. Such downregulation suggested that the surplus glycolysis intermediates were potentially redirected toward the shikimate pathway or other metabolic pathways (Fig. 4). Substantiating this proposition, significant elevations were observed in both SA and its derivatives (2,3-DHBA and 2,5-DHBA) (Fig. 4). Additionally, a strikingly higher accumulation was detected in 14 out of 15 identified flavonoids, with 12 identified glycosides also falling under the classification of flavonoids (Fig. 4, Table S7). The metabolome results mentioned above demonstrate the crucial regulatory role of *Sl*TOR in directing the glycolysis and shikimate pathways toward amino acids or SA and flavonoids.

Upon *Sl*TOR inactivation at 4°C, there was an increased accumulation of Put and citrulline, both of which are derivatives of glutamate (Fig. 4). This led to a reduction in glutamine levels, which also originated from glutamate (Fig. 4). Put is the predominant polyamine found in higher plants. Previous studies have highlighted the significance of polyamines in the plant’s response to various environmental stresses [37]. On the other hand, citrulline serves as an intermediate in Put biosynthesis. Our findings indicate that *Sl*TOR plays a pivotal role in determining the allocation of glutamate to stress-related Put or amino acid glutamine. Additionally, the compounds aspartate, threonine, and lysine exhibited a notable decrease following treatment with Torin2 at 4°C, indicating suppression of amino acid biosynthesis by *Sl*TOR inhibition (Fig. 4). Collectively, our metabolome analysis implied a sophisticated trade-off between amino acid metabolism and the development of cold tolerance, regulated by *Sl*TOR.

### *Sl*TOR mediated metabolic changes by reprogramming the transcriptome

Cold stress induces a significant transcriptional reprogramming to promote the accumulation of cryoprotective metabolites [18]. Our results show that *Sl*TOR regulates numerous cold-responsive genes, particularly *SlCBF1*, a key player in cold responses, as depicted in Fig. 1j–k, and Fig. 2d–e. Consequently, we hypothesized that TOR could potentially influence the metabolic alterations by controlling the expression of genes encoding pivotal enzymes involved in metabolite synthesis.

Under 4°C, Torin2 treatment resulted in the increased accumulation of 3-PGA and PEP. This was further supported by the examination of the expression profiles of key regulatory genes involved in the upstream reactions of 3-PGA and PEP production. Specifically, we analyzed the expression patterns of *SlPFK2* (*PHOSPHOFRUCTOKINASE 2*, Solyc04g015200.3), *SlPFK7* (*PHOSPHOFRUCTOKINASE 7*, Solyc07g045160.3), *SlGAPC* (*CYTOSOLIC GLYCERALDEHYDE-3-PHOSPHATE DEHYDROGENASE*, Solyc01g098950.3), and *SlPGH1* (also known as *CYTOSOLIC ENOLASE2*, *ENO2*, Solyc09g009020.3). These genes encode enzymes responsible for catalyzing crucial steps in the conversion of substrates such as Fructose-6-phosphate to Fructose-1,6-bisphosphate, Glyceraldehyde-3-phosphate to 1,3-Bisphosphoglycerate, and 2-Phosphoglycerate to PEP, which are essential reactions leading to 3-PGA and PEP synthesis. Notably, upon Torin2 treatment at 4°C, a significant upregulation in the expression levels of *SlPFK2*, *SlPFK7*, *SlGAPC*, and *SlPGH1* was observed (Fig. 4).

Next, we examined the expression levels of genes involved in the shikimate pathway and SA biosynthesis. CHORISMATE SYNTHASE 2 (*Sl*CS2, Solyc04g009620.5) catalyzes the final reaction of the shikimate pathway to form chorismite; and ISOCHORISMATE SYNTHASE 1 (*Sl*ICS1, Solyc06g071030.3) converts chorismate to isochorismate, initiating the SA biosynthesis process [36]. Notably, TOR inhibitor treatment at 4°C led to increased expression levels of both *SlCS2* and *SlICS1* (Fig. 4). The expression patterns of these SA synthesis genes align well with the observation that Torin2 treatment at 4°C promoted the accumulation of SA (Fig. 4 and Table S5).

Due to the accumulation of citrulline, we detected the transcript level of tomato *ORNITHINE TRANSCARBAMOYLASE* (*SlOTC*, Solyc04g080610.3), encoding the enzyme converting ornithine into citrulline within the Put synthesis pathway, in response to TOR dysfunction and chilling stress [41]. Torin2 induced *SlOTC* transcripts during cold treatment. Besides, *ARGININE DECARBOXYLASE 1* and *2* (*SlADC1*, Solyc01g110440.5 and *SlADC2*, Solyc10g054440.3) encode catalytic enzymes involved in the decarboxylation of arginine into agmatine within the Put synthesis pathway [48]. The expression of *SlADC1* and *SlADC2* was also induced by a combined *Sl*TOR inhibition and cold stress (Fig. 4). These findings emphasize that *Sl*TOR plays a significant role in regulating the transcription of genes encoding key enzymes, leading to Put accumulation.

Meanwhile, as inhibition of TOR under 4°C reduced the content of alanine, aspartate, glutamine, serine, and lysine and threonine produced from aspartate (Fig. 4), we also tested the expression patterns of genes encoding the key enzymes in alanine, aspartate, glutamine, and serine biosynthesis, including tomato *ALANINE AMINOTRANSFERASE 2* (*SlAlaAT2*, Solyc03g123600.4) for alanine synthesis, *ASPARTATE AMINOTRANSFERASE 1* and *5* (*SlAspAT1*, Solyc07g055210.3 and *SlAspAT5*, Solyc08g041870.3) for aspartate synthesis, *GLUTAMINE SYNTHASE 1;1* (*SlGLN1;1*, Solyc04g014510.3) for glutamine synthesis, and *GLYCERATE DEHYDROGENASE* (*SlGDH*, Solyc01g111630.3) and *SERINE HYDROXYMETHYLTRANSFERASE 4* (*SlSHM4*, Solyc12g098490.2) for serine biosynthesis [43–45]. As expected, Torin2 treatment did reduce the expressions of *SlGLN1* under 4°C, which agrees with the metabolite profile (Fig. S7). Interestingly, the same treatments did not affect the expressions of aspartate and serine synthesizing genes, or even induced the alanine synthesizing gene (Fig. S7), indicating that TOR might regulate the biosynthesis of these amino acids at post-transcriptional levels.

**Figure 5:**
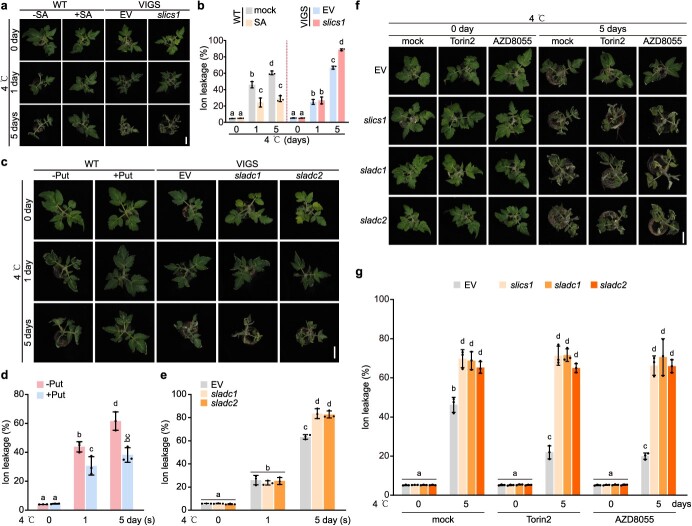
SA and Put enhanced tomato cold tolerance. (a) SA-treated (1 mM) 4-week-old tomato plants showed elevated cold tolerance 1 day and 5 days post-4°C cold treatment compared with mock-treated plants, while silencing *SlICS1* by VIGS (*slics1*) led to susceptibility to 4°C cold treatment 5 days post-treatment. Representative images were captured. Scale bar = 2 cm. (b) Quantification analysis of ion leakage of (a). Data were analyzed from three experiments, and expressed as mean ± SD (c) Put-treated (1 mM) 4-week-old tomato plants showed elevated cold tolerance 1 and 5 days post-4°C cold treatment compared with mock-treated plants, while silencing *SlADC1* and *SlADC2* by VIGS (*sladc1* and *sladc2*) led to susceptibility to 4°C cold treatment 5 days post-treatment. Representative images were captured. Scale bar = 2 cm. (d) Quantification analysis of ion leakage of 4-week-old tomato plants with or without Put under 4°C treatment at indicated times. Data were analyzed from 3 experiments, and expressed as mean ± SD (e) Quantification analysis of ion leakage in control lines (EV) and *sladc1* or *sladc2* VIGS lines under 4°C treatment at indicated times. (f) Silencing *SlICS1*, *SlADC1*, and *SlADC2* by VIGS (*slics1*, *sladc1*, and *sladc2*) led to susceptibility to 4°C cold treatment 5 days post-treatment compared with control plants (EV). Two-hour Torin2 or AZD8055 pretreatment enhanced EV cold tolerance, but did not increase the cold susceptibility of *slics1*, *sladc1*, and *sladc2* VIGS lines. Representative images were captured. Scale bar = 2 cm. (g) Quantification analysis of ion leakage of (f). Data were analyzed from three experiments, and expressed as mean ± SD. Statistical significance was determined by one-way ANOVA with Tukey’s *post hoc* test. Lowercase letters indicate statistically significant differences between the mean values (*P* < 0.05).

Our results revealed that the metabolic changes induced by Torin2 <4°C were mostly consistent with the expression patterns of genes encoding crucial enzymes for biosynthesis. Specifically, deviations were observed in the expression of amino acid biosynthesis genes, indicating the potential involvement of post-transcriptional regulations mediated by *Sl*TOR.

### SA and Put accumulation upon TOR inhibition promoted tomato cold tolerance

Substantial enrichment of SA and Put was observed in *Sl*TOR-suppressed tomato plants during cold stress (Fig. 4). SA and Put have already been known for their crucial physiological functions in cold stress responses [38–40]. To further evaluate the impact of SA and Put on tomato cold tolerance, we conducted gain- and loss-of-function analyses to estimate the capacity of these metabolites in enhancing cold tolerance in tomato. Pretreating tomato plants with 1 mM SA or Put before subjecting them to 4°C for 1 or 5 days reduced wilting and cell death compared to the mock control plants, indicating improved cold tolerance (Fig. 5a and 5c). Additionally, virus-induced gene silencing (VIGS) was employed to target the *SlICS1*, *SlADC1*, and *SlADC2* genes (Fig. S8). During a 1-day cold treatment, blocking either the SA or Put biosynthesis pathway in VIGS lines did not affect the plants’ cold responses, potentially due to functional redundancy (Fig. 5a and 5c). However, the VIGS-*SlICS1*-silenced (*slics1*), *SlADC1*-silenced (*sladc1*), and *SlADC2*-silenced (*sladc2*) lines exhibited significant foliar chlorosis and cell death phenotypes compared to the VIGS-empty vector (EV) control after 5 days of cold treatment (Fig. 5a and 5c). This indicates that during a prolonged 5-day cold treatment resulting in more severe damage, both pathways are critical for cold tolerance. The observed differences in cell death phenotypes were further supported by the assessment of cell viability through electrolyte leakage analysis (Fig. 5b, 5d, and 5e).

Furthermore, treatment with Torin2 or AZD8055 did not improve the susceptibility of *slics1*, *sladc1*, and *sladc2* to the 5-day cold treatment, suggesting that *Sl*TOR acts upstream of these crucial enzymes in the biosynthesis of SA or Put (Fig. 5f–g).

### *Sl*TOR may induce transcriptome and subsequent metabolome reprogramming through *Sl*PGH1

To investigate how *Sl*TOR regulates the transcriptome and subsequent metabolome reprogramming, we performed a yeast two-hybrid (Y2H) library screen using the C-terminus of *Sl*TOR containing the kinase domain as a prey to explore *Sl*TOR-interacting proteins. During this investigation, we discovered that *Sl*PGH1 is a *Sl*TOR-interacting protein. *Sl*PGH1 is the homolog of *At*ENO2 (CYTOSOLIC ENOLASE2, At2G36530) in *Arabidopsis* (Fig. S9). It is worth noting that *At*ENO2 plays a critical role in transcriptionally activating *At*CBF1, a central transcription factor in plants [42].

**Figure 6:**
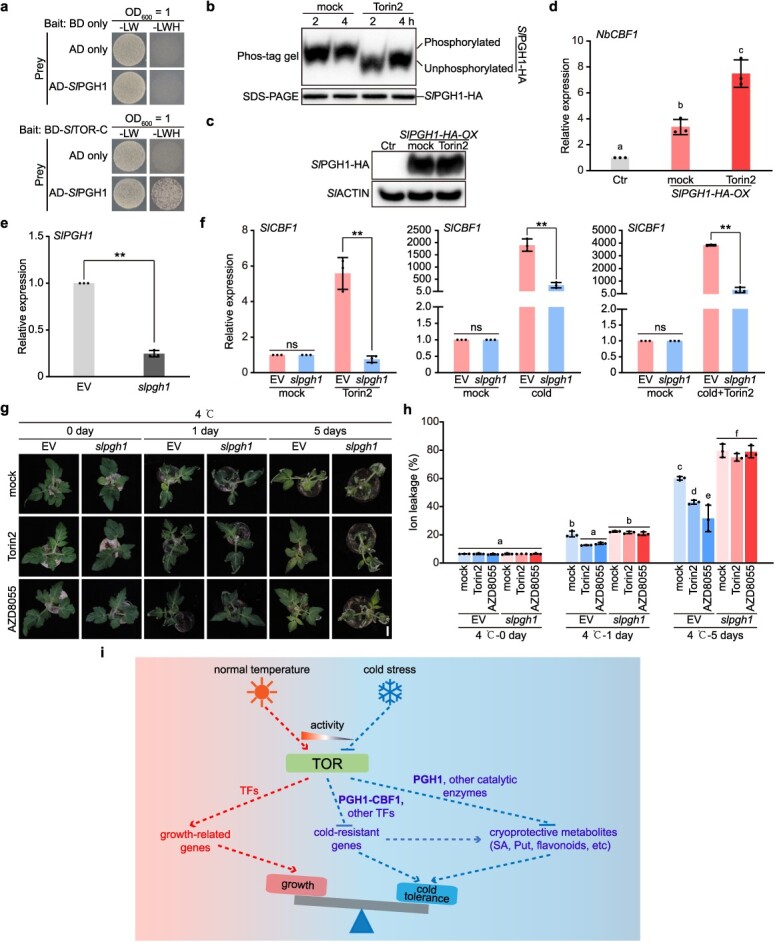
*Sl*TOR modulates *CBF1* expression through *Sl*PGH1. (a) *Sl*TOR interacts with *Sl*PGH1, as revealed by Y2H analyses. L, Leu; W, Trp; H, His. (b) Torin2 treatment caused a significant *Sl*PGH1-HA band shift, as observed through phos-tag gel analysis. *35S::SlPGH1-HA* were transiently expressed in tobacco leaves. Proteins extracted from leaves treated with either Torin2 or mock for different durations were used for immunoblotting, separated by phos-tag gel or normal SDS page gel. Two independent biological replicates showed similar results. (c) *35S::SlPGH1-HA* were transiently expressed in tobacco leaves. Untransfected leaves were used as a control (Ctr). Tobacco leaves overexpressing *SlPGH1-HA* were treated with or without 10 μM Torin2 for 2 h. The expression of *Sl*PGH1-HA was detected by immunoblotting. *Sl*ACTIN were used as loading control. (d) RT-qPCR analysis of the transcript levels of *NbCBF1* in tobacco leaves overexpressing *SlPGH1*. *NbEF1α* (AF120093.1) was used as an internal standard. RT-qPCR was performed using RNA isolated from tobacco leaves. Mean ± SD, *n* = 3 biological replicates. (e) RT-qPCR analysis of the transcript levels of *SlPGH1* in VIGS line (*slpgh1*) and control line (EV). Mean ± SD, *n* = 3 biological replicates. *SlACTIN7* was used as an internal standard. (f) RT-qPCR analysis of the transcript levels of *SlCBF1* in VIGS-*SlPGH1*-silenced line (*slpgh1*) and control line (EV) under 3-h cold, Torin2, or cold plus Torin2 treatment. Mean ± SD, *n* = 3 biological replicates. *SlACTIN7* was used as an internal standard. (g) Silencing *SlPGH1* by VIGS (*slpgh1*) led to susceptibility to 4°C cold treatment 5 days post-treatment compared with control plants (EV). Two-hour Torin2 or AZD8055 pretreatment enhanced EV cold tolerance, but did not increase the cold susceptibility of *slpgh1* VIGS lines. Representative images were captured. Scale bar = 2 cm. (h) Quantification analysis of ion leakage of (g). Data were analyzed from three experiments, and expressed as mean ± SD. For (d) and (h), statistical significance was determined by one-way ANOVA with Tukey’s *post hoc* test. Lowercase letters indicate statistically significant differences between the mean values (*P* < 0.05). For (e) and (f), the asterisk indicates statistically significant differences between the mean values of different treatments (**, *P* < 0.01, Student’s *t*-test). (i) A work model for TOR-mediated balance between growth and cold tolerance. Under normal temperatures, active TOR induces growth-promoting genes, enhancing growth. Cold stress inhibits TOR activity, activating cold-responsive genes and producing cryoprotective metabolites. The TOR-PGH1-CBF1 axis plays a crucial role in transcriptome reprogramming. Additionally, *Sl*TOR-induced phosphorylation of *Sl*PGH1 and other enzymes may influence metabolic alterations.

We confirmed the interaction between *Sl*TOR-C and *Sl*PGH1 in yeast cells (Fig. 6a). The TOR kinase in tobacco (*Nicotiana benthamiana*) and tomato exhibits an extremely high degree of conservation (94% identity). To evaluate the inhibitory effect of the TOR inhibitor Torin2 on *Nb*TOR kinase activity, we transiently expressed *AtS6K1* in tobacco leaves and determined that 10 μM Torin2 efficiently inhibited *Nb*TOR’s activity (Fig. S10a–b). Meanwhile, 10 μM Torin2 significantly inhibited tobacco seedling growth (Fig. S10c–d). Taking advantage of the high identity of TOR between tomato and tobacco, we conducted experiments by transiently overexpressing *SlPGH1* in tobacco leaves to investigate whether *Nb*TOR phosphorylates *Sl*PGH1 *in vivo*, utilizing phos-tag gel analysis as a detection method. The results showed that Torin2 treatment induced a significant band shift of *Sl*PGH1 in the phos-tag gel analysis, indicating *Sl*PGH1 is a potential substrate of *Sl*TOR (Fig. 6b).

To investigate the impact of TOR-induced phosphorylation on the expression of *CBF1*, we initiated the experiment by transiently overexpressing *SlPGH1* in tobacco leaves (Fig. 6c). Subsequently, the tobacco leaves were subjected to a 2-h treatment of Torin2 before examining the expression of *NbCBF1* (EU727155.1) utilizing quantitative reverse transcription PCR (RT-qPCR). The findings revealed that the overexpression of *SlPGH1* led to an augmentation in *NbCBF1* expression, which was further enhanced by Torin2 treatment (Fig. 6c–d). Following this observation, the *SlPGH1* gene was silenced in tomato through VIGS (Fig. 6e). An evaluation of *SlCBF1* expression in the VIGS line unveiled that the silencing of *SlPGH1* resulted in reduced *SlCBF1* expression under cold, Torin2, and cold plus Torin2 treatments (Fig. 6f). The VIGS-*SlPGH1*-silenced line exhibited increased sensitivity to cold treatment, with no alleviation in phenotype upon Torin2 treatment (Fig. 6g–h). This result elucidated that the *Sl*TOR-*Sl*PGH1 axis played a significant role in mediating the cold response of tomatoes.

The collective outcomes highlight the influence of the *Sl*TOR-*Sl*PGH1 link in regulating *CBF1* expression, a pivotal transcription factor in cold responses. These results suggest that *Sl*TOR could potentially trigger transcriptome and subsequent metabolome reprogramming through the modulation of *Sl*PGH1.

## Discussion

As a central regulator in plant signaling network, TOR precisely balances the competing demands of growth and defense [2, 3]. We explored the *Sl*TOR-regulated transcription network in tomato, revealing that TOR promotes growth-related processes, while concurrently inhibiting stress responses (Fig. 1j). These findings support the notion that TOR acts as a pivotal switch governing the transition between growth and defense.

TOR inhibition activates numerous cold-regulated genes, suggesting its involvement in regulating cold stress responses (Fig. 1k). *Sl*TOR activity is promptly and temporarily suppressed by cold stress within a mere 1-h timeframe; and with prolonged exposure to cold conditions, TOR activity rebounds, potentially due to a compensatory mechanism aimed at preventing adverse effects associated with prolonged TOR suppression (Fig. S2b-c). Although cold stress transiently inhibits TOR activity, further inhibition of TOR under cold conditions, enhances tomato cold tolerance (Fig. 2, Fig. S2d-g).

Plants have developed various advanced strategies, including metabolic regulation, to reduce cold-induced damage [46]. Although TOR has been indicated to play a vital role in metabolomics reprogramming [4, 6, 7, 27–31], the regulatory role of TOR at the metabolic level under stress conditions is poorly understood. We checked the metabolic alternations induced by *Sl*TOR under cold stress. Notably, we discovered a tight link between TOR activity and amino acid metabolism under cold treatment. TOR activity and amino acid accumulation are intricately connected under normal growth conditions. Most branches of amino acids could function upstream to activate *At*TOR [11]. Meanwhile, TOR inhibition also causes extensive accumulation of amino acids [6, 27, 30, 47]. However, certain amino acids, such as aromatic amino acids, proline, and arginine, cannot activate TOR, but connect to plant stress responses [49]. In cold-treated *Sl*TOR-repressed plants, we observe a downregulation of amino acids that typically activate TOR, including alanine, aspartate, glutamine, lysine, serine, and threonine [11] (Fig. 4). In contrast, derivatives from aromatic amino acids and arginine (SA, Put, and flavonoids) that contribute to cold tolerance are highly accumulated. (Fig. 4). Therefore, we deduce that *Sl*TOR could conduct the flow direction of amino acid biosynthesis toward different downstream metabolites under diverse environmental conditions.

In addition to the metabolic changes, the inactivation of *Sl*TOR significantly mediates the expression of genes encoding catalytic enzymes involved in glycolysis, SA and Put biosynthesis, and amino acids biosynthesis (Fig. 4). The expression patterns align well with the metabolic alterations, pointing to a potential mechanism in which *Sl*TOR modulates the phosphorylation state of critical transcriptional regulators to mediate the amino acid-derived metabolism (Fig. 4). Based on this speculation, we uncovered a potential substrate of *Sl*TOR, a transcription factor known as *Sl*PGH1. *Sl*PGH1 is a direct homolog of *At*ENO2 in *Arabidopsis*, which is encoded by the *AtLOS2* (At2G36530) gene [42]. *AtLOS2* produces two independent proteins through different translation initiation sites: the 444 AA *At*ENO2 and the 352 AA *At*MBP-1. *Sl*PGH1 is homologous to *At*ENO2 but not to *At*MBP-1 [42] (Fig. S9). *AtENO2* has been reported to activate the transcription of *AtCBF1* in *Arabidopsis* [42]. The *Sl*TOR-*Sl*PGH1 link has been shown to govern *CBF1* expression, which is a fundamental transcription factor crucial for plant cold responses. This finding indicates the significant involvement of the *Sl*TOR-*Sl*PGH1-*Sl*CBF1 axis in initiating extensive transcriptome reprogramming and subsequent metabolic changes. Interestingly, we also observed the induction of *SlPGH1* transcription upon TOR inhibition at 4°C (Fig. 4). Additionally, *At*ENO2 is essential for glycolysis, serving as a key enzyme that catalyzes the conversion of 2-Phosphoglycerate to PEP [42]. *Sl*TOR-induced phosphorylation of *Sl*PGH1 may directly influence *Sl*PGH1’s catalytic activity. Inhibiting this phosphorylation might redirect carbon flux into the downstream shikimate pathway, producing cryoprotective metabolites, although the detailed mechanism remains to be explored.

Additionally, given the apparent decoupling between the accumulation of amino acids and the transcripts of genes encoding amino acid biosynthetic enzymes (Fig. 4 and Fig. S7), it is also possible that *Sl*TOR may also phosphorylate some catalytic or regulatory components in amino acid-related metabolic pathways to direct metabolic fluxes toward specific downstream metabolites.

We proposed a model to summarize our findings on the TOR-regulated trade-off between growth and cold tolerance (Fig. 6i).

Future research endeavors ought to focus on elucidating the specific mechanisms through which *Sl*TOR regulates this equilibrium, particularly by identifying more substrates targeted by *Sl*TOR kinase. Such investigations are likely to provide valuable insights into the intricate regulatory mechanisms governing the trade-offs between plant growth and stress responses.

## Materials and methods

### Plant materials and growth conditions

Sterilized Micro-Tom tomato seeds were grown on solid 1/2 Murashige and Skoog (MS) medium in a growth chamber (Jiupu, China) under long-day (LD) conditions (16 h light/8 h dark, 300 μmol·m^−2^·s^−1^ of light intensity, 25 ± 1°C) for 7 days. The 7-day-old seedlings were then transplanted into Jiffy-7 pellets (Jiffy Products International AS, Norway) and grown in a glasshouse under LD conditions (300–400 μmol·m^−2^·s^−1^ of light intensity, 60%–70% relative humidity, 25°C ± 1°C). Four-week-old plants were used for 4°C treatment in a growth chamber, under similar conditions.

### Generation of transgenic plants, and transient expression in tobacco leaves

For plant expression vectors, the coding sequence (CDS) of *AtS6K1* and *SlPGH1* (without stop codons) were PCR amplified and were inserted into a modified pCAMBIA1300 plant expression vector, including a 3xHA tag driven by the CaMV 35S promoter. For inducible RNAi gene silencing, a 315-bp fragment of *SlTOR* CDS was amplified and inserted into the pBWA(V)HVE vector. The cloning was performed using the ClonExpress II One Step Cloning Kit (Vazyme, C112). Primers are listed in Table S1.

The vectors were transformed into *Agrobacterium tumefaciens* (GV3101) using the freeze–thaw method. Transgenic plants were produced in WT tomatoes through the *Agrobacterium*-mediated leaf disk method. The transgenic plants were selected with the hygromycin B resistance and were confirmed through PCR amplification of the resistance gene fragment (Table S1). For transient expression in tobacco, *Agrobacterium* cell cultures with OD_600_ = 0.5 in infiltration buffer (10 mM MES, pH = 5.6, 10 mM MgCl_2_) were injected into leaves of 5-week-old tobacco.

### Chemical treatments

Tomato seeds were geminated in solid 1/2 MS medium containing Torin2 (Selleck Chemicals, S2817) or AZD8055 (Selleck Chemicals, S1555) for 7 days to check TOR inhibitors’ effects on seedling growth. Four-week-old plants were treated with 10 μM Torin2, 10 μM AZD8055, 1 mM SA (Sigma-Aldrich, S7401), or 1 mM Put (MedChemExpress, HY-N2407) through foliar spray. For *tor-es* inducible RNAi lines, *tor-es* seeds were germinated in solid 1/2 MS medium with 10 μM estradiol (Merck, E2785) for 7 days to check TOR’s function in tomato seedlings. Additionally, 4-week-old *tor-es* plants were watered with 10 μM estradiol 1 week before cold treatment to silence *TOR*.

### RNA extraction and RT-qPCR

Total RNA was isolated with the Total RNA Extraction Reagent (Vazyme, R401). 1 μg RNA was used for reverse transcription with the HiScript II 1st Strand cDNA Synthesis Kit (Vazyme, R212). RT-qPCR was performed using the AceQ Universal SYBR qPCR Master Mix (Vazyme, Q511) on a qTOWER3 real-time PCR thermal cycler (Analytik Jena).

### Protein extraction and immunoblot analysis

The plant tissue was ground in liquid nitrogen. Total protein was isolated from the powdered tissue with a 2xSDS sample buffer containing 80 mM Tris–HCl (pH 6.8), 2% (w/v) SDS, 10% (v/v) glycerol, 3% (v/v) 2-Mercaptoethanol, and 0.1% (w/v) bromophenol blue. The total proteins were separated by SDS-PAGE and were then blotted onto Immobilon-PSQ PVDF membrane (Millipore, ISEQ00010). The *At*S6K1-HA or *Sl*PGH1-HA protein was detected through immunoblotting using anti-HA antibody (1:5000; ABclonal, AE008). The phospho-p70 S6 kinase (Thr389) antibody (1:2000; Cell Signaling Technology, 9205) was used to monitor the phosphorylation status of Thr449 in *At*S6K1. *Sl*ACTIN was detected by anti-ACTIN antibody (1:5000; ABclonal, AC009). Additionally, phos-tag gel analysis was conducted following the manufacturer’s instructions (Vazyme, PA101).

### RNA-seq and statistical analysis

Four biological replicates of 10-day-old tomato seedlings, with or without 5-μM Torin2 treatment, were used for RNA extraction using the Total RNA Extraction Reagent (Vazyme, R401). The RNA-seq and analysis were performed by OE Biotech Co., Ltd. (Shanghai, China) following standard procedures. Briefly, RNA-Seq libraries were constructed with the VAHTS Universal V6 RNA-seq Library Prep Kit, and the sequencing was performed on an Illumina Novaseq 6000 platform, generating 150-bp paired-end reads. The high-quality reads were then aligned to the tomato reference genome (SL4.0, https://solgenomics.net) using HISAT2 software. EdgeR was used to explore DEGs, with the significance cutoffs of | log2 fold change (FC) | > 1 and FDR < 0.05. Next, GO enrichment analysis of these DEGs was performed using R (v 3.2.0) to identify significantly enriched terms, based on the hypergeometric distribution.

### Physiological measurements and histochemical staining

To test electrolyte leakage, the third and fourth true leaves from the tomato plants were collected at specified time points and immersed in 30 ml of deionized water. After a 30-min vacuum treatment, the leaves were incubated at room temperature for 4 h. The conductivity of the solution was tested both before and after boiling with a conductivity meter (INESA, DDSJ-307F). For each measurement, four leaves per treatment were utilized, and three replicates were conducted. Additionally, the levels of H_2_O_2_ and O_2_^• –^ were quantified with specific kits (Solarbio, BC3590 and BC1290), following the manufacturer’s guidelines.

The histochemical detection of H_2_O_2_ and O_2_^• –^ was performed using diaminobenzidine (DAB) and nitroblue tetrazolium (NBT), respectively. For DAB staining, leaf samples were collected and immersed in a DAB staining solution (1 mg/ml, pH 3.8). Following a 30-min vacuum treatment, the samples were incubated at room temperature overnight. Next, the stained leaves were transferred to 95% ethanol and incubated in a water bath at 80°C for 10 min to facilitate destaining. For NBT staining, leaf samples were placed into an NBT staining solution (1 mg/ml in 50 mM phosphate buffer, pH 7.4) and subjected to a 30-min vacuum. The samples were then stained for another 2 h. Then, the leaf samples were decolorized in 95% ethanol. The destained leaves upon DAB staining or NBT staining were preserved in a 25% (v/v) glycerol solution for documentation.

### Metabolomic profiling and statistical analysis

The widely targeted metabonomic analysis was performed by Bioprofile Biotechnology Co., Ltd (Shanghai, China) following standard protocol. Briefly, the metabolites were isolated from plant tissues with 1 ml methanol/acetonitrile/water (v/v, 2:2:1) under sonication for 1 h. The resulting extracts were then used for liquid chromatography-mass spectrometry (LC–MS) analysis. Metabolites with a *P* < 0.05 and a VIP > 1 were considered as DAMs. A KEGG pathway analysis was performed using these DAMs to identify the perturbed biological pathways. KEGG enrichment analyses were performed through Fisher’s exact test and FDR correction for multiple testing. A significance threshold of *P* < 0.05 was applied to discover significantly enriched KEGG pathways.

### VIGS

VIGS was performed as previously described [50], with minor modifications. Briefly, 300-bp fragments of the CDS from *SlICS1*, *SlADC1*, *SlADC2*, and *SlPGH1* were PCR amplified and cloned into the VIGS vector pTRV2 using the ClonExpress II One Step Cloning Kit (Vazyme, C112). The primers are listed in Table S1. These pTRV2 constructs were transformed into the *A. tumefaciens* strain GV3101. The *Agrobacterium* strains containing these pTRV2 vectors were combined with the strain containing the VIGS vector pTRV1 at a 1:1 ratio. This mixture was then infiltrated into the cotyledons of 2-week-old tomato seedlings. The seedlings were then subject to 2-day dark treatment before being grown in a glasshouse. Seedlings that were infiltrated with the pTRV2 empty vector and pTRV1 were used as a negative control.

### Yeast two-hybrid analysis

For Y2H vectors, *SlTOR-C* and *SlPGH1* coding sequences were PCR amplified and inserted into the pGBKT7 and pGADT7 vectors using the ClonExpress II One Step Cloning Kit (Vazyme, C112). The Y2H library screening was conducted by OE Biotech Co., Ltd. (Shanghai, China) following standard protocol. The Y2H to confirm protein–protein interaction was performed as described [10].

### Statistical analysis

For comparisons involving two groups, Student’s *t*-test was employed, utilizing Microsoft Excel. In cases where more than two samples were compared, one-way analysis of variance (ANOVA) with *post hoc* Tukey’s HSD test was performed using GraphPad Prism 8.

### Accession numbers

The RNA-seq raw data for the *Sl*TOR-regulated transcriptome have been deposited in the NCBI Gene Expression Omnibus (GEO; https://www.ncbi.nlm.nih.gov/geo/) under the accession number: GSE266850. The raw data for widely targeted metabolomics have been deposited in the MetaboLights (https://www.ebi.ac.uk/metabolights/) under the accession number: MTBLS10015.

## Supplementary Material

Web_Material_uhae253
