# Supplementary material for: TOR balances plant growth and cold tolerance by orchestrating amino acid-derived metabolism in tomato
Source: Hortic Res. 2024 Sep 5;11(12):uhae253. doi: 10.1093/hr/uhae253 (PMC11630258; doi:10.1093/hr/uhae253)
Supplement: Web_Material_uhae253 [file web_material_uhae253.zip › 04-Supplementary Material Figs. S1-S10.docx]

**Fig. S1**


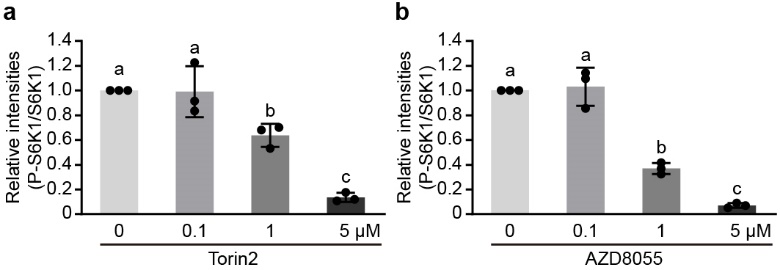


**Fig. S1 Quantification analysis of TOR activity under TOR inhibitor treatments.** (a) and (b) Quantification of relative P-T449 intensity of Fig. 1f and Fig. 1i. Mean ± s.d., n = 3 biological replicates. Statistical significance was determined by one-way ANOVA with Tukey’s post-hoc test. Lowercase letters indicate statistically significant differences between the mean values (*p* ＜ 0.05).

**Fig. S2**


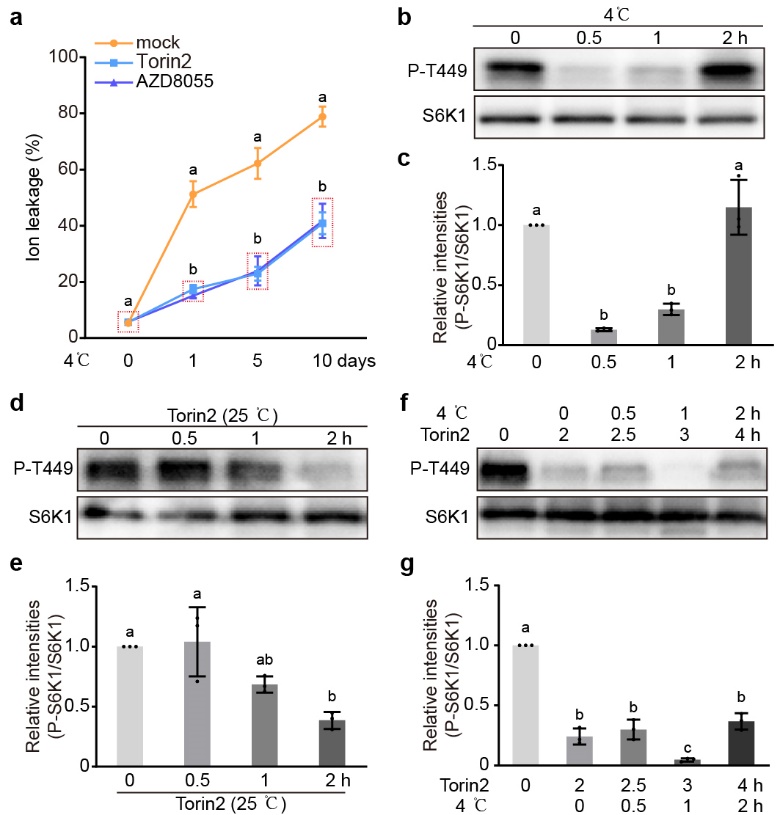


**Fig. S2 Inhibition of TOR enhanced tomato cold tolerance and cold alters TOR activity.** (a) Quantification of ion leakage with or without TOR inhibitor pretreatment at indicated times under 4 ℃ cold treatment. Mean ± s.d., n = 3 biological replicates. (b) Western blot analysis of *Sl*TOR kinase activity in *35S::AtS6K1-HA*/Micro-Tom transgenic seedlings, based on phosphorylation of Thr449 on S6K1 (P-T449, a specific phosphorylation site of TOR kinase on S6K1) after treatment with indicated times of cold treatment. (c) Quantification of relative P-T449 intensity of (b). Mean ± s.d., n = 3 biological replicates. (d) and (f) Torin2 treatment under 25 ℃ (d) or 4 ℃ (f) through foliar spray effectively inhibited TOR activity. (e) and (g) Quantification of relative P-T449 intensity of (d) and (f), respectively. Mean ± SD, n = 3 biological replicates. Statistical significance (a), (c), (e), and (g) was determined by one-way ANOVA with Tukey’s post-hoc test. Lowercase letters indicate statistically significant differences between the mean values (*p* ＜ 0.05).

**Fig. S3**


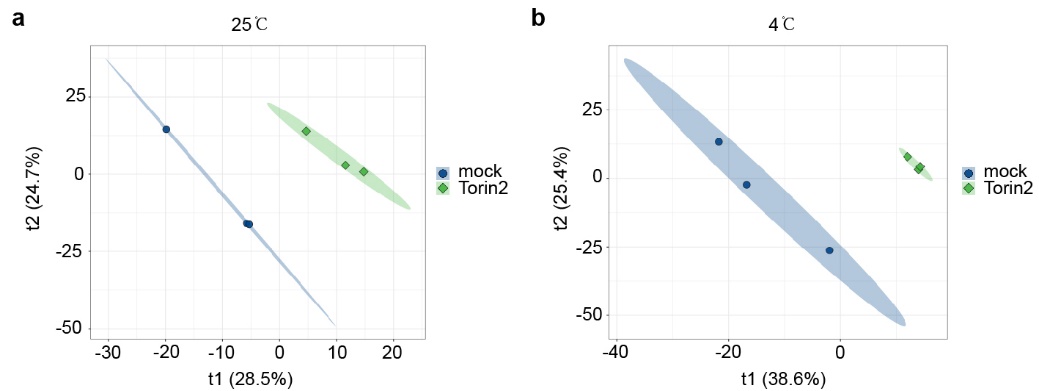


**Fig. S3 A partial least squares discriminant analysis (PLS-DA) of the metabolomic profiles between Torin2-treated and mock-treated samples at 25 ℃ (a) and 4 ℃ (b).**

**Fig. S4**


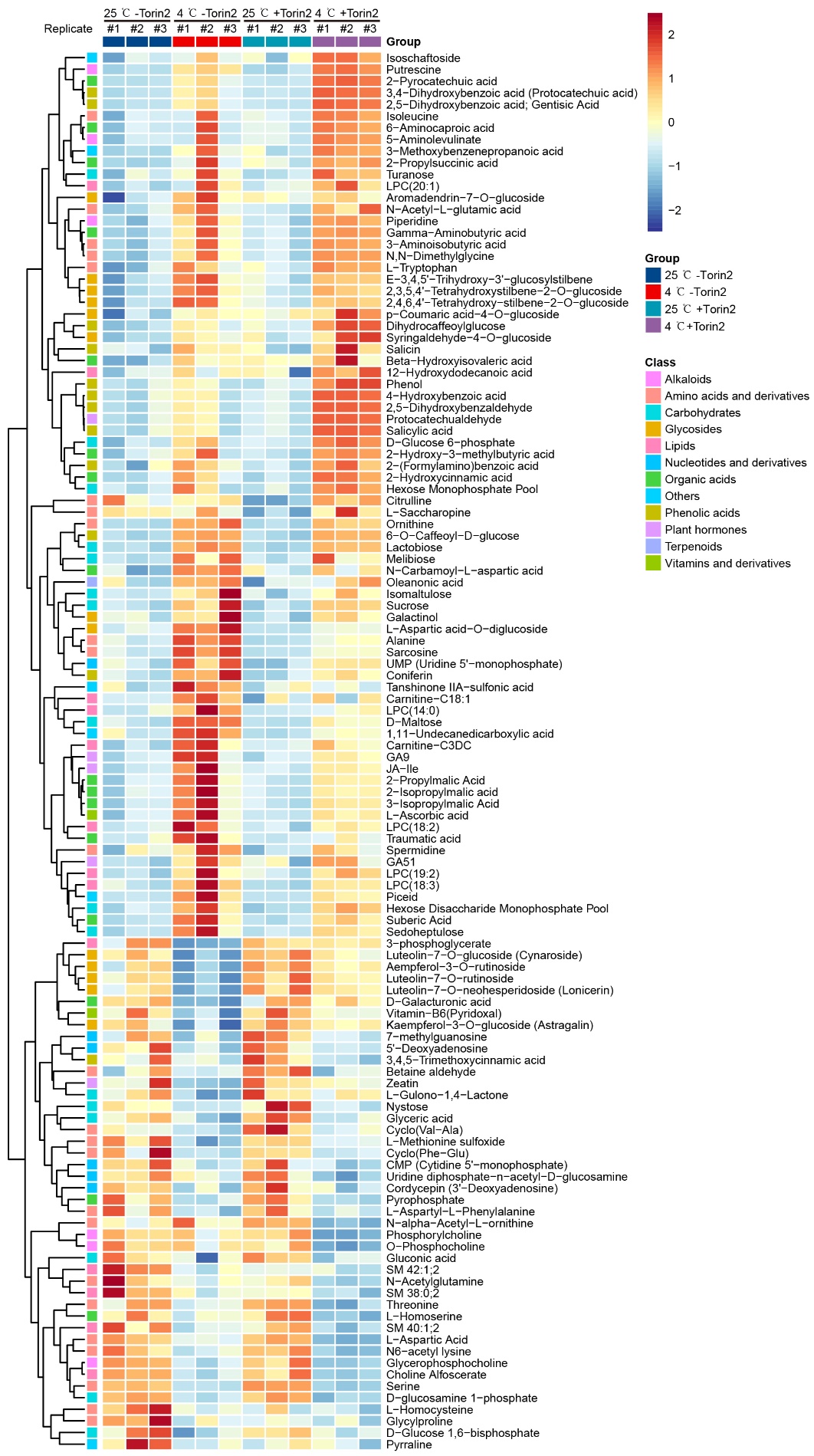


**Fig. S4 Widely targeted metabolomics analysis reveals contrasting accumulation of metabolites between mock and Torin2-treated tomato plants at 25 ℃ or 4 ℃.** The heat map illustrates DAMs between mock and Torin2-treated tomato plants at 25 ℃ or 4 ℃. Each row represents a single metabolite. The scored intensity ranges from red (+2.0) through white (0) to blue (-2.0). Different colours in “Group” indicate different treatments. Different colours in “Class” indicate different classes of metabolites.

**Fig. S5**


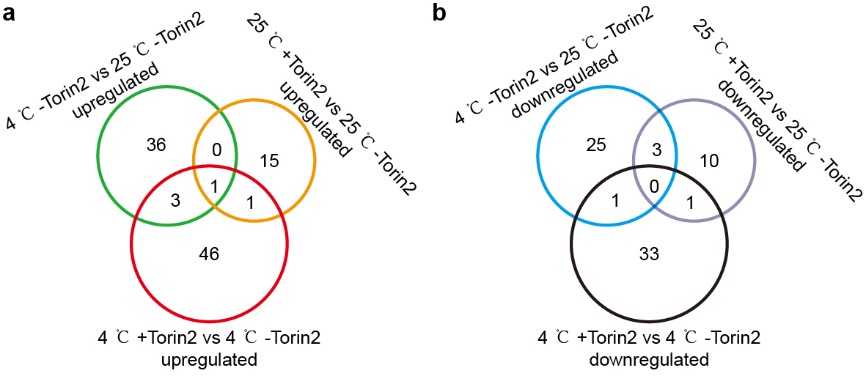


**Fig. S5 Comparisons of DAMs under different treatments.** (a) Comparisons of upregulated DAMs among 4 ℃ -Torin2 vs 25 ℃ -Torin2, 25 ℃ +Torin2 vs 25 ℃ -Torin2, and 4 ℃ +Torin2 vs 4 ℃ -Torin2. (b) Comparisons of downregulated DAMs among 4 ℃ -Torin2 vs 25 ℃ -Torin2, 25 ℃ +Torin2 vs 25 ℃ -Torin2, and 4 ℃ +Torin2 vs 4 ℃ -Torin2.

**Fig. S6**


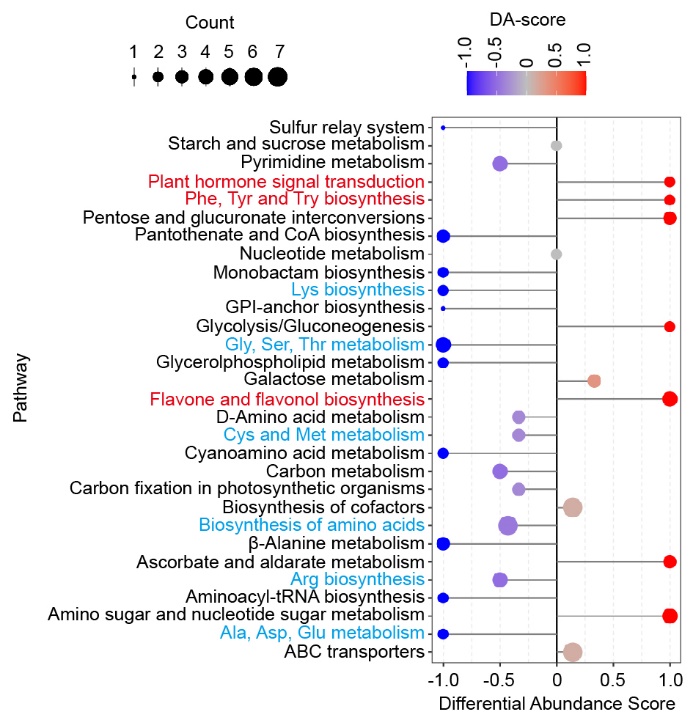


**Fig. S6 Differential abundance score (DA score) of TOP 30 enriched metabolic pathways in Torin2-treated tomato plants at 4 ℃ compared to mock-treated plants.** The size of the dot indicates the number of DAMs annotated in the pathway. DA score reflects the overall changes of all metabolites in the metabolic pathway. A score of 1.0 indicates an upward trend in the abundance of all annotated metabolites in the pathway. In contrast, -1.0 indicates a downward trend in the abundance of all annotated metabolites in the pathway. A score closer to 1.0 or -1.0 indicates that the overall abundance of all metabolites in this pathway tends to be upregulated or downregulated. The length of the line segment represents the absolute value of the DA score.

**Fig. S7**


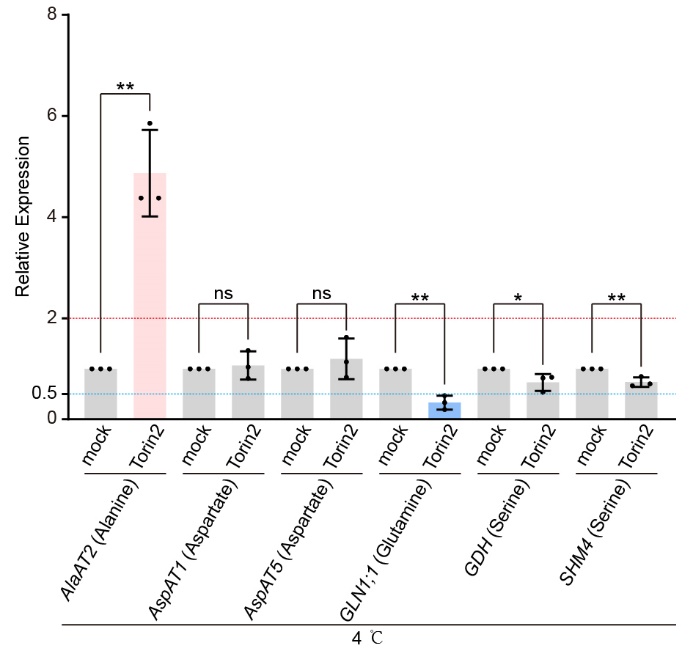


**Fig. S7 RT-qPCR analysis of the transcript levels of synthesizing genes for different amino acids with or without Torin2 treatment under 4 ℃.** Mean ± s.d., n = 3 biological replicates. *SlACTIN7* was used as an internal standard. Statistical significance was determined by two-sided Student’s t-test. **, *p* ＜ 0.01; *, *p* ＜ 0.05; ns, not significant.

**Fig. S8**


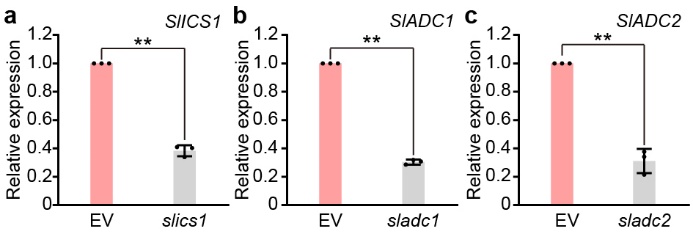


**Fig. S8 RT-qPCR analysis of the transcript levels of *SlICS1* (a), *SlADC1* (b), and *SlADC2* (c) in VIGS lines.** Mean ± s.d., n = 3 biological replicates. *SlACTIN7* was used as an internal standard. The asterisk indicates statistically significant differences between the mean values of different treatments (**, *p* < 0.01, Student’s *t*-test).

**Fig. S9**


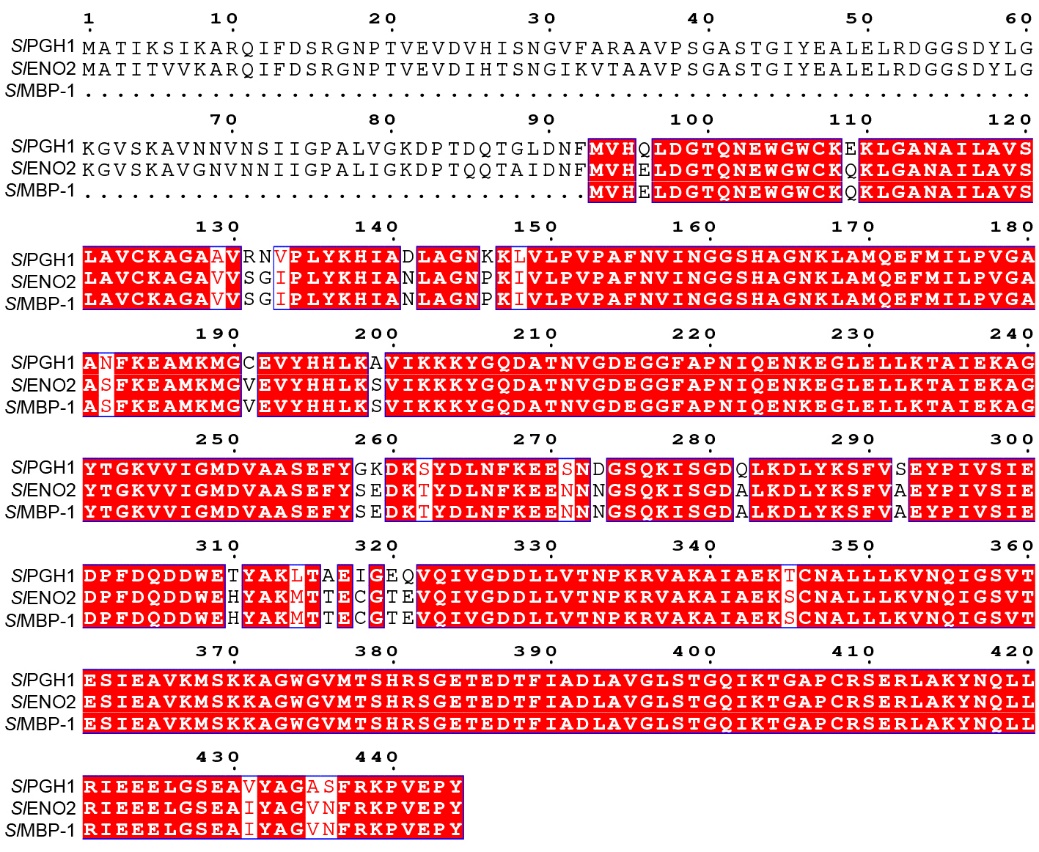


**Fig. S9 Sequence alignment of *Sl*PGH1, *At*ENO2, and *At*MBP-1.**

**Fig. S10**


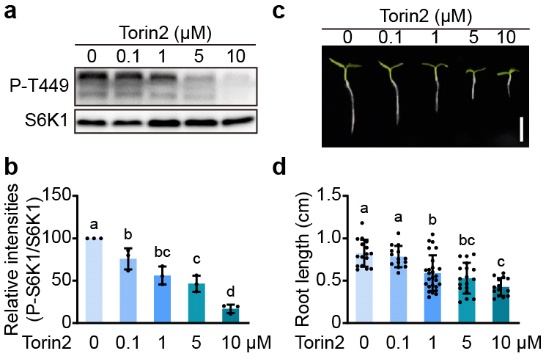


**Fig. S10 Torin2 inhibits TOR activity in tobacco.** (a) Torin2 inhibits *Nb*TOR activity. Western blot analysis of *Nb*TOR kinase activity in tobacco leaves transiently expressing *AtS6K1-HA*, based on phosphorylation of Thr449 on S6K1 (P-T449, a specific phosphorylation site of TOR kinase on S6K1) after treatment with indicated concentrations of Torin2. (b) Quantification of relative P-T449 intensity of (a). Mean ± s.d., n = 3 biological replicates. Statistical significance was determined by one-way ANOVA with Tukey’s post-hoc test. Lowercase letters indicate statistically significant differences between the mean values (*p* ＜ 0.05). (c) Torin2 retarded tobacco seedling growth. Tobacco seeds were germinated on 1/2 MS medium with different concentrations of Torin2 for 7 days. Scale bars = 0.5 cm. (d) Quantifications of the root length of (c). Data were analyzed from 10-30 seedlings for each treatment, and expressed as mean ± s.d. Statistical significance in (b) and (d) was determined by one-way ANOVA with Tukey’s post-hoc test. Lowercase letters indicate statistically significant differences between the mean values (*p*＜0.05).
