# Supplementary figures and images for: TOR balances plant growth and cold tolerance by orchestrating amino acid-derived metabolism in tomato
Source: Hortic Res. 2024 Sep 5;11(12):uhae253. doi: 10.1093/hr/uhae253 (PMC11630258; doi:10.1093/hr/uhae253)

Fig. 1d

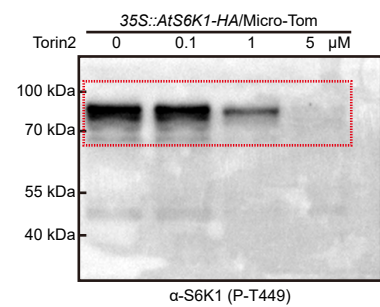

Fig. 1g

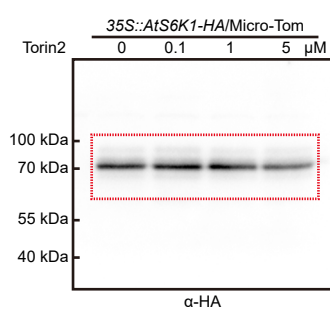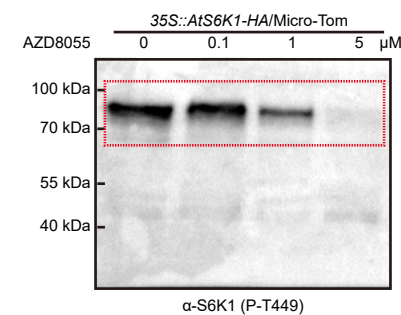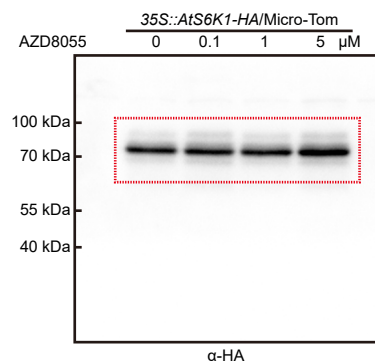

Fig. 6b

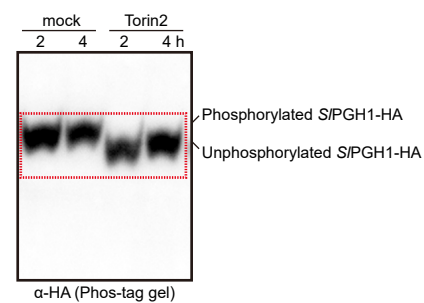

Fig. 6b

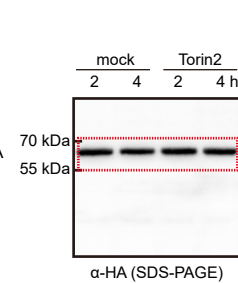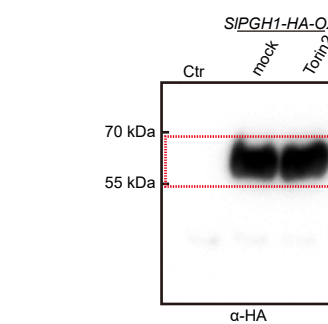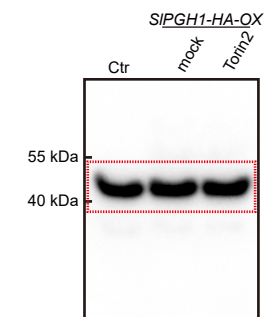

Fig. S2b

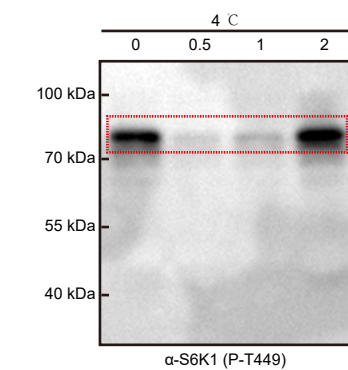

Fig. S2d

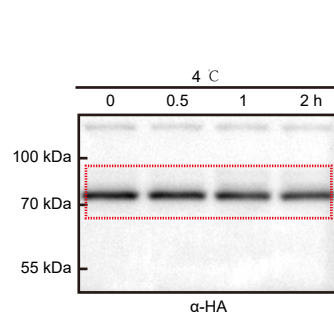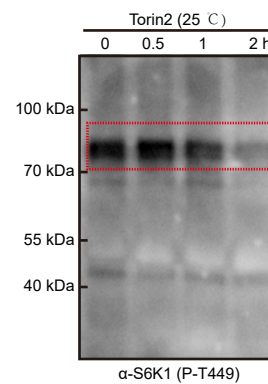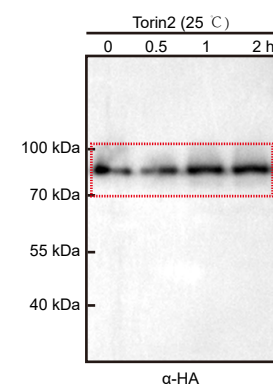

Fig. S2f

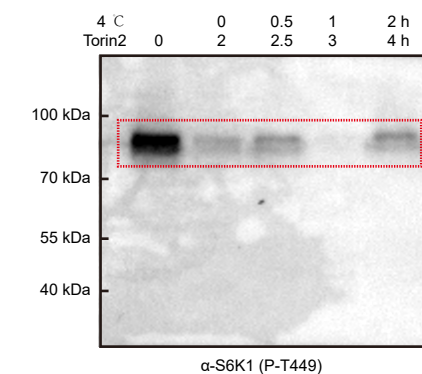

Fig. S10a

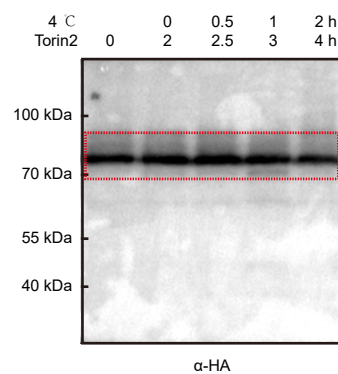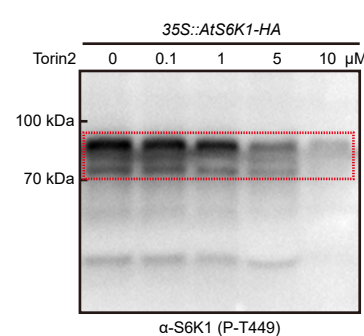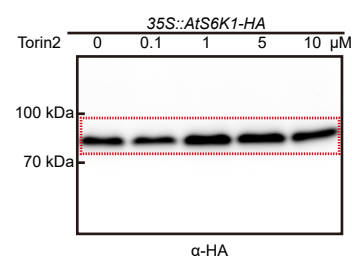

Supplement: Web_Material_uhae253 [file web_material_uhae253.zip › 07-Supplementary Material gel source data.pdf]
